# Leverage points for resilience: Introducing the pyramid framework for strategic adaptation planning and assessment

**DOI:** 10.1007/s13280-025-02216-7

**Published:** 2025-07-24

**Authors:** Linda Rosengren, Karuna Budhathoki, Juho Haapala, Katriina Soini, Mila Sell

**Affiliations:** 1 Natural Resources Institute Finland (Luke), Latokartanonkaari 9, 00790 Helsinki, Finland; 2 Viikki Tropical Resources Institute (VITRI), University of Helsinki, Latokartanonkaari 7, 00790 Helsinki, Finland; 3 NIRAS Finland, Tikkurila, Vantaa 01300 Finland; 4 Aalto University, Espoo, Finland; 5 Natural Resources Institute Finland (Luke), Survontie 9A, 40500 Jyväskylä, Finland

**Keywords:** Food system transformation, Leverage points, Nepal, Resilience

## Abstract

Sustainability transformation is a rapidly evolving field, yet few studies have explored approaches for food system transformation. This study addresses that gap by designing and applying a pyramid framework that integrates the leverage point perspective with three resilience capacities: coping, adaptive, and transformative. The goal of the framework is to (1) shed light on root causes that shape resilience and (2) pinpoint effective entry points for strengthening resilience and driving transformation. Using qualitative methods, this study applied the framework to a food system in western Nepal, identifying eleven leverage points. Results showed that deep leverage points supported transformative capacity, while shallow ones reinforced coping and adaptive capacities. The pyramid framework balances immediate shock responses with long-term planning. This integrated approach mitigates trade-offs between resilience strategies, reducing maladaptation risks. The pyramid framework serves as a tool for ex-ante strategic adaptation planning and ex-post assessment and evaluation of resilience policy and interventions.

## Introduction

Food systems worldwide face complex, interconnected challenges related to sustainability, including environmental degradation, food insecurity, inequitable access to productive resources, and shifting dietary patterns. There is broad consensus among both scientific (IPBES 2019; IPCC 2023) and policy institutions (European Union 2019; FAO 2022; World Bank 2024) that resilience is essential in tackling these challenges and that transforming food systems is essential to enhance sustainability. Research on this subject has grown rapidly as shown in an extensive review spanning three decades (Juri et al. 2024).

Resilience is defined as a system’s capacity to withstand disruptive change while maintaining its core functions (Olsson et al. 2014). This capacity is often conceptualised as comprising coping, adaptive, and transformative capacities (Folke 2010), each of which is equally critical to resilience (Béné et al. 2012). This depiction has been widely adopted in resilience research (Berman et al. 2012; Arnall 2015; Masik and Gajewski 2021; Turchi et al. 2023).

While existing research on system transformation has provided valuable insights into the factors and processes that drive change, few studies propose heuristic tools for identifying effective entry points for strengthening all three resilience capacities (coping, adaptive, and transformative capacity). The 3R approach (Zurek et al. 2022) and the adaptive waves approach (Luthe and Wyss 2015) do consider the three resilience capacities but do not offer a guidance for identifying strategic entry points to strengthen them. The transformative adaptation research alliance’s (TARA) approach (Colloff et al. 2017) integrates adaptation and transformation within governance and institutional change but lacks an explicit focus on coping capacity. The so-called ABCD resilience assessment framework (Fonteijn 2022) does not explicitly consider transformative aspects.

The objective of this study is to design and apply the so-called pyramid framework presented in detail in Sect. "Theoretical framework". The framework aims to uncover the root causes shaping resilience, identify effective entry points to strengthen coping, adaptive, and transformative capacity simultaneously, and map transformative pathways. The leverage points perspective has been widely used as a heuristic tool for identifying effective intervention strategies that promote desired change, particularly in sustainability transformations (Chan et al. 2020; Leventon et al. 2021; Riechers et al. 2021). Its relevance is particularly pronounced when studying complex systems such as food systems and has gained traction among both researchers and practitioners (Dorninger et al. 2020; Rosengren et al. 2020; Zimmermann et al. 2023; Wopereis et al. 2024).

This study is based on qualitative data collected in western Nepal, specifically in the Sudurpaschim and Karnali provinces. Unlike the more developed regions of Nepal, western Nepal remains geographically remote and culturally distinct (Adhikari and Gellner 2024). Nepal provides a compelling context for studying resilience and system transformation as the country’s new constitution devolves extensive decision-making authority to the local level, specifically to municipalities (Chaudhary 2019). Additionally, Nepalese communities have a strong tradition of self-organisation, which supports locally relevant decision-making and leadership. Western Nepal is particularly suitable for transformation research as it is currently undergoing fundamental socio-ecological changes driven by road development, market expansion, high labour migration due to limited local opportunities, and climate change impacts on food security (Bhandari 2018; Tome et al. 2022).

This study is novel in that it explicitly applies the leverage points perspective to simultaneously identify entry points for strengthening the three resilience capacities in the context of a food system. Furthermore, the study contributes to the operationalisation of transformation, an area with significant knowledge gaps (Fedele et al. 2019; Salomaa and Juhola 2020; Sousa et al. 2024) and to the currently limited body of research on transformation in the Global South (Asadzadeh et al. 2022).

## Theoretical framework

This study builds on two key concepts—leverage points and resilience—both of which originate from systems thinking. While these concepts address different aspects of systems research, they share a common focus on system change. Leverage points are defined as “*places within a system where a small shift may lead to a fundamental change in the system as a whole*” (Abson et al. 2017), making them particularly relevant for sustainability research. Resilience is commonly conceptualised as a combination of coping, adaptive, and transformative capacities (Walker et al. 2004; Folke 2010). Accordingly, resilience is not an outcome but rather the agency and capability of individuals, communities, institutions, or nations to make decisions on how they respond to change (Béné et al. 2016). While these capacities are treated as distinct in this study, they often overlap and exist along a continuum.

Coping capacity refers to a system’s short-term ability to absorb shocks and maintain function without undergoing major structural changes. Adaptive capacity involves incremental adjustments that extend beyond coping but do not shift the system’s stability domain. Transformative capacity—also referred to as transformative resilience (Asadzadeh et al. 2022) or transformational resilience (Bahadur and Tanner 2014)—describes a system’s ability to undergo fundamental changes that address the root causes of stressors, enabling a shift to a new stability domain. It is considered an emergent property influenced by place and context, agency, and interaction (Wolfram 2016), with innovation playing a significant role (Hölscher et al. 2019). Transformative capacity has been defined as "*the capacity of individuals and organizations to transform themselves and their societies in a deliberate, conscious way*" (Ziervogel et al. 2016).

The leverage points perspective builds on Donella Meadows’ (1999) original concept of the 12-point leverage framework. Abson et al. (2017) later grouped these 12 points into four realms: intent, design, feedback, and material. The intent realm, the so-called “deepest” level of the leverage point framework, addresses worldviews, values, and paradigms that shape society and drive system change. These deep leverage points are essential for achieving lasting, transformative shifts by tackling the root causes of systemic functions. The design realm pertains to the structural and organisational elements that determine how a system operates, including rules, policies, and configurations that influence behaviour. The feedback realm focuses on mechanisms within a system that either amplify and accelerate behaviours (positive feedback) or slow down changes (negative feedback). Feedback mechanisms are crucial in regulating system dynamics. The material realm, considered the “shallowest” level of the framework, concerns system stocks and flows, including specific numbers, targets, thresholds, and other measurable quantities that govern system management.

**Fig. 1:**
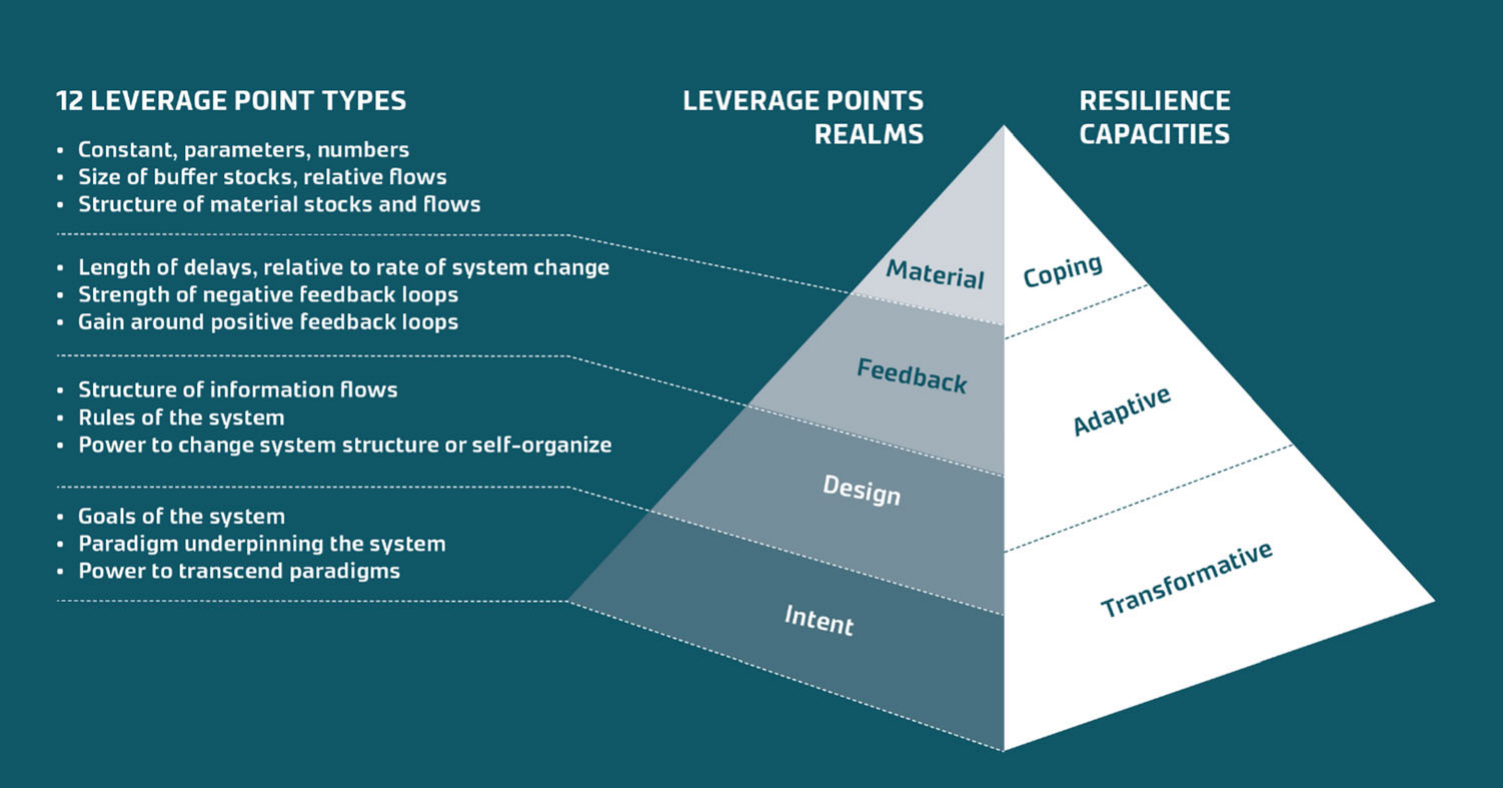
The pyramid framework, which combines the four leverage point realms with the three resilience capacities

When the leverage points framework is placed beside the depiction of the three resilience capacities by Folke (2010), a similar logic emerges. Figure 1 visualises this with the help of the so-called pyramid framework. We can see that the lowest part of the pyramid, representing the intent realm, aligns with transformation-related considerations such as structures, norms, worldviews, and values. The top of the pyramid, featuring the material realm, aligns with coping capacity, as access to necessary resources is crucial for coping after a natural disaster. This study aims to assess whether this analogy holds and if the pyramid framework can provide a useful approach for understanding the root causes that shape resilience, pinpoint strategic entry points for strengthening resilience, and identify pathways for food system transformation.

## Materials and methods

### Case study area of western Nepal

**Fig. 2:**
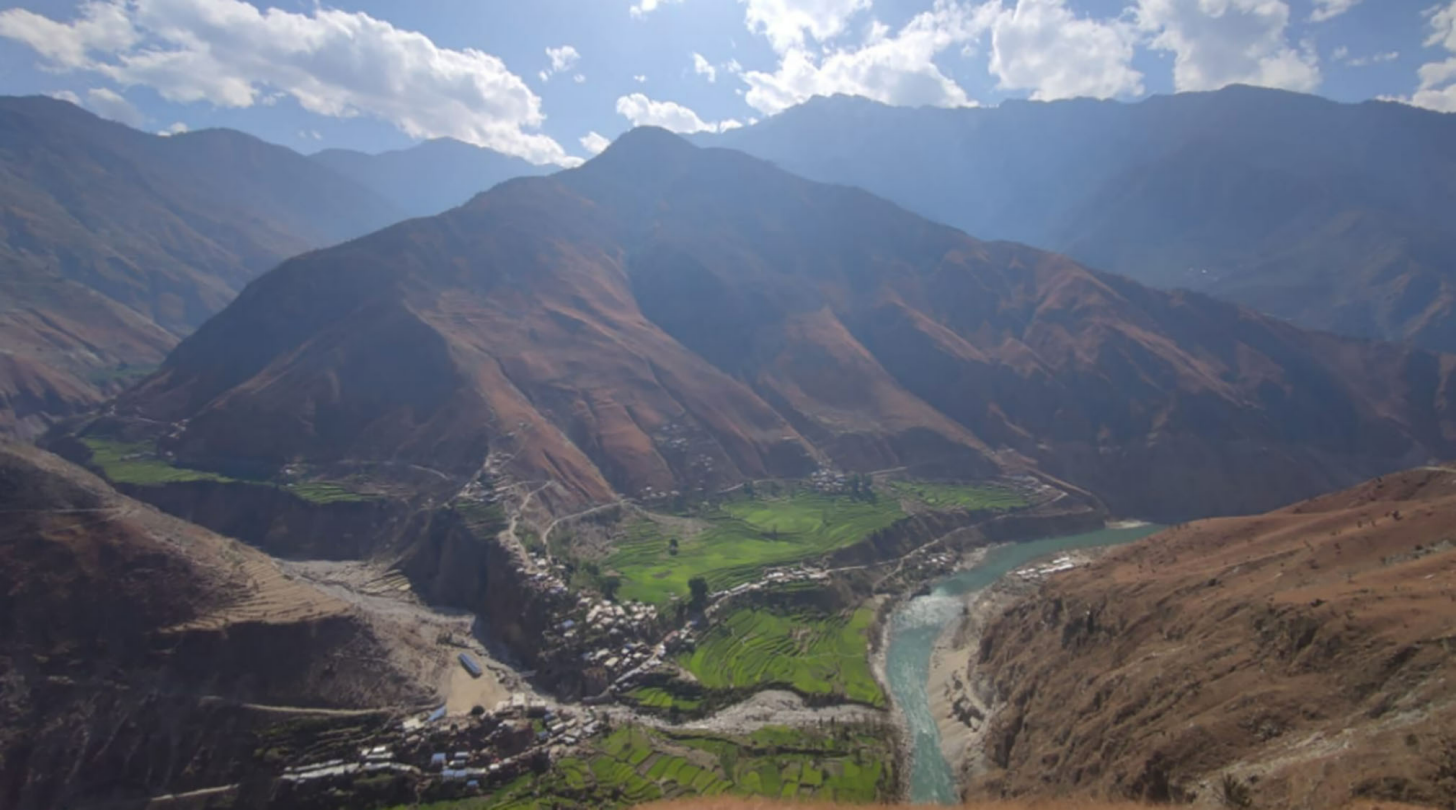
Landscape in Sinja Municipality, Western Nepal. Photo by: Juho Haapala

The study area comprises five districts in the Karnali and Sudurpaschim provinces in Western Nepal. These represent the two most remote, rural, and least developed provinces of Nepal according to, e.g. Multidimensional Poverty Index (Government of Nepal 2021b) and several other indicators (Government of Nepal 2021a) (Fig. 2).

Western Nepal is currently undergoing rapid change, which we briefly outline below. Climate change is significantly impacting Nepal’s food system, particularly through unpredictable weather patterns and increased natural disasters including floods (Joshi and Bhandari 2022). Crop yields are declining due to temperature changes and the spread of pests, and water scarcity is making irrigation more challenging (Risal et al. 2022). These challenges are particularly severe for marginalised communities, who have less access to resources. Efforts to adapt, such as promoting climate-resilient crop varieties and improving water management, are, therefore, crucial to building a more sustainable and resilient food system in Nepal.

Nepal has recently implemented important changes in its governance system, some of which directly impact the Nepalese food system. A new constitution from 2015 devolves power to local governments over areas such as education, health, infrastructure, and local taxation, promulgated by the Local Government Operation Act 2017 (Chaudhary 2019). Local governments now have greater authority to develop and implement policies tailored to the specific agricultural needs of their regions, including decisions on land use, irrigation, and local agricultural development programmes. While this decentralisation allows for more responsive and context-specific interventions, the resulting effectiveness is dependent on the capacity and resources of local governments, which vary across regions, and factors such as technical and administrative adeptness, available resources, collaborative networks, and political incorruptibility (Acharya 2018).

Another important driver shaping food systems in western Nepal is the gradual transition to a market economy, facilitated by infrastructure development including roads and improved telecommunication networks. The emerging market economy is promoting commercialisation, further infrastructure development, and changing consumption patterns (Gc and Hall 2020).

**Fig. 3:**
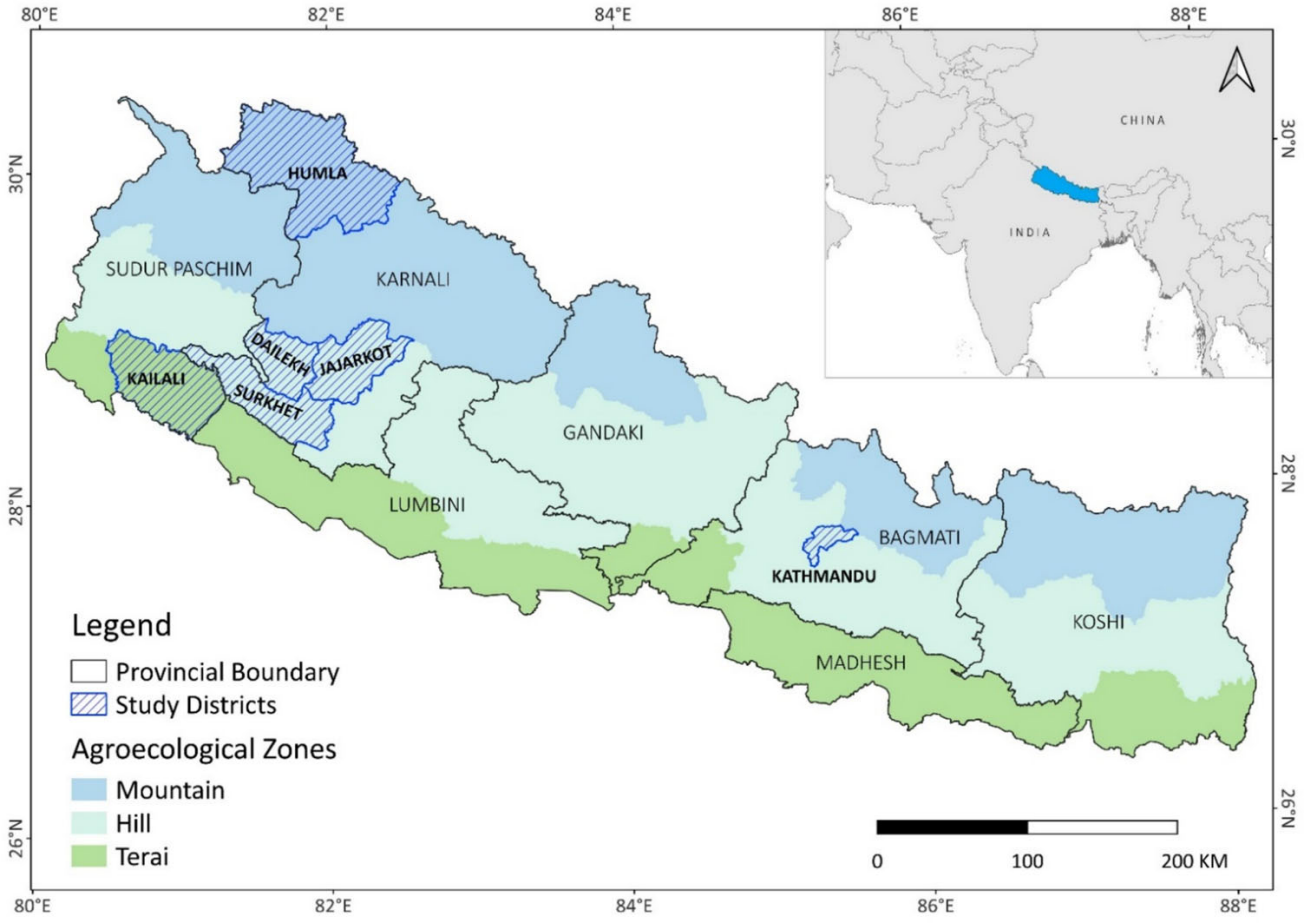
Map of Nepal and the three agro-ecological zones. Districts where data was collected are marked with blue stripes. The districts represent all three agroecological zones of Nepal: Humla is one of the remotest areas of Nepal, located in the high mountains and lacking, e.g., a reliable road access, whereas Jajarkot, Dailekh and Surkhet are in the Midhills representing a typical forest covered hilly area, dotted with small family farms. Kailali lays in the lowland of Terai, having a tropical climate, clearly bigger population density and much better access to markets and services.

Large-scale migration from Nepal, especially to India and the Gulf countries, driven by limited local job opportunities in western Nepal has become central to the country’s socio-economic landscape (Shrestha 2017). Remittances from migrants have reduced poverty, funded education, and improved living standards back home, but they have also shifted family dynamics, with many households now being led by women or elderly members (Chaudhary 2020; Sunam 2020). This migration has also caused agricultural labour shortages, increasing fallow land, and reducing productivity, with some rural villages potentially becoming uninhabitable if trends continue, as seen in India’s Himalayan region (Rai 2022; Kumar and Misra 2024) (Fig. 3).

### Sampling and data collection

The study applied a qualitative research methodology, collecting data through key informant interviews (*n* = 39) and focus group discussions (*n* = 2). We used purposive sampling (Tongco 2007) to identify research participants representing three major actor groups: experts, government officials, and farmers. We conducted in-person semi-structured interviews in six districts: Kathmandu, the capital of the federal nation; Kailali, a Terai, lowland district from Sudurpaschim: Surkhet, Dailekh, and Jajarkot, three Midhill districts from Karnali; and Humla, a mountain district from Karnali (Fig. 3). Interviews in the capital were conducted with experts to obtain national perspectives, while interviews in the districts provided contextual local viewpoints. In addition, we collected data from two focus group discussions with farmers in Surkhet and Dailekh. The groups consisted of ten purposively sampled men and women.

The interviews and focus group discussions addressed various aspects of the food system, including questions on different types of food production systems, land tenure, food security, access to finance and information, aspects relating to the food value chain, social organisation, values and attitudes towards farming, and food and food system drivers. Questions covered overall trends, policy, and governance arrangements of these topics. The questions were tailored to the three actor groups (farmer, government official, and expert).

### Data analysis

All the interviews were recorded with the consent of the participants. The audio files were later transcribed and translated into English language. The transcripts were analysed using thematic analysis (Braun 2019) in the NVivo software. We coded against the broad categories of the interview script including a description of the food system, main drivers of change, challenges, and recommendations for improvement. When identifying the leverage points presented in the Results (Sect. "Results"), we considered research participants’ views and suggestions that intersected with the leverage point framework and the three capacities of resilience: coping, adaptive, and transformative capacity (see Fig. 1).

## Results

Below we present the leverage points identified through the application of the pyramid framework in western Nepal. The results are organised around the four realms of the leverage point framework: intent, design, feedback, and material.

### Addressing system goals and paradigms—intent realm

Based on the interviews and focus group discussions, we identified and contextualised three leverage points from the intent realm, featuring the deepest realm addressing worldviews, values, and system paradigms. These cover stronger food self-sufficiency, a value shift towards taking pride in the local, traditional food and farming culture, as well as a shift in the existing paradigms related to patriarchal and discriminating values shaping the food system.

#### Leverage point 1: Dynamic and connected rural areas supporting food self-sufficiency

Low food self-sufficiency makes Nepal vulnerable to shocks. In western Nepal, particularly in the Midhills and High Mountain areas, the food system is predominantly based on subsistence farming. Due to limited arable land, many households can only produce enough food for a few months each year, leading to an increasing reliance on imported foods, primarily rice from India. Efforts have been made to commercialise crops such as vegetables, but market inefficiencies continue to hinder significant progress. In contrast, the Terai lowlands, with greater arable land and better access to services, have a more developed market economy, with evolved value chains that foster small- to medium-scale farms and food businesses.

Although full food self-sufficiency may not be feasible or desirable for Nepal, increasing it would enhance both food system resilience and food security. The Terai, with its fertile land, is well-suited for staple crop production, while the Midhills offer favourable conditions for vegetable cultivation. As key informant 06 states: "*We consider that Terai is the source of the food grains and that feeds the hill and mountains*". Improving self-sufficiency and food security would require deliberate efforts to enhance agricultural efficiency. This entails adopting less labour-intensive methods, which reduce the workload of farmers while maintaining essential levels of production.

Leverage Point 1 (dynamic and connected rural areas supporting food self-sufficiency) is interpreted as reinforcing both coping and transformative capacity. Within the broader ambition of revitalising rural areas, Leverage Point 1 represents a reframing of system goals, aligning with the intent realm (Fig. 1). From this perspective, it promotes social organisation and cohesion while encouraging anticipatory future planning, thereby supporting transformative capacity (Table 1, see row “Intent”). Conversely, when viewed through the lens of food self-sufficiency, this leverage point enhances the system’s stock of food, serving as a safeguard against trade disruptions and climate shocks. In this context, it primarily supports coping capacity and aligns with the material realm (Table 1, see row “Material”).

**Table 1:**
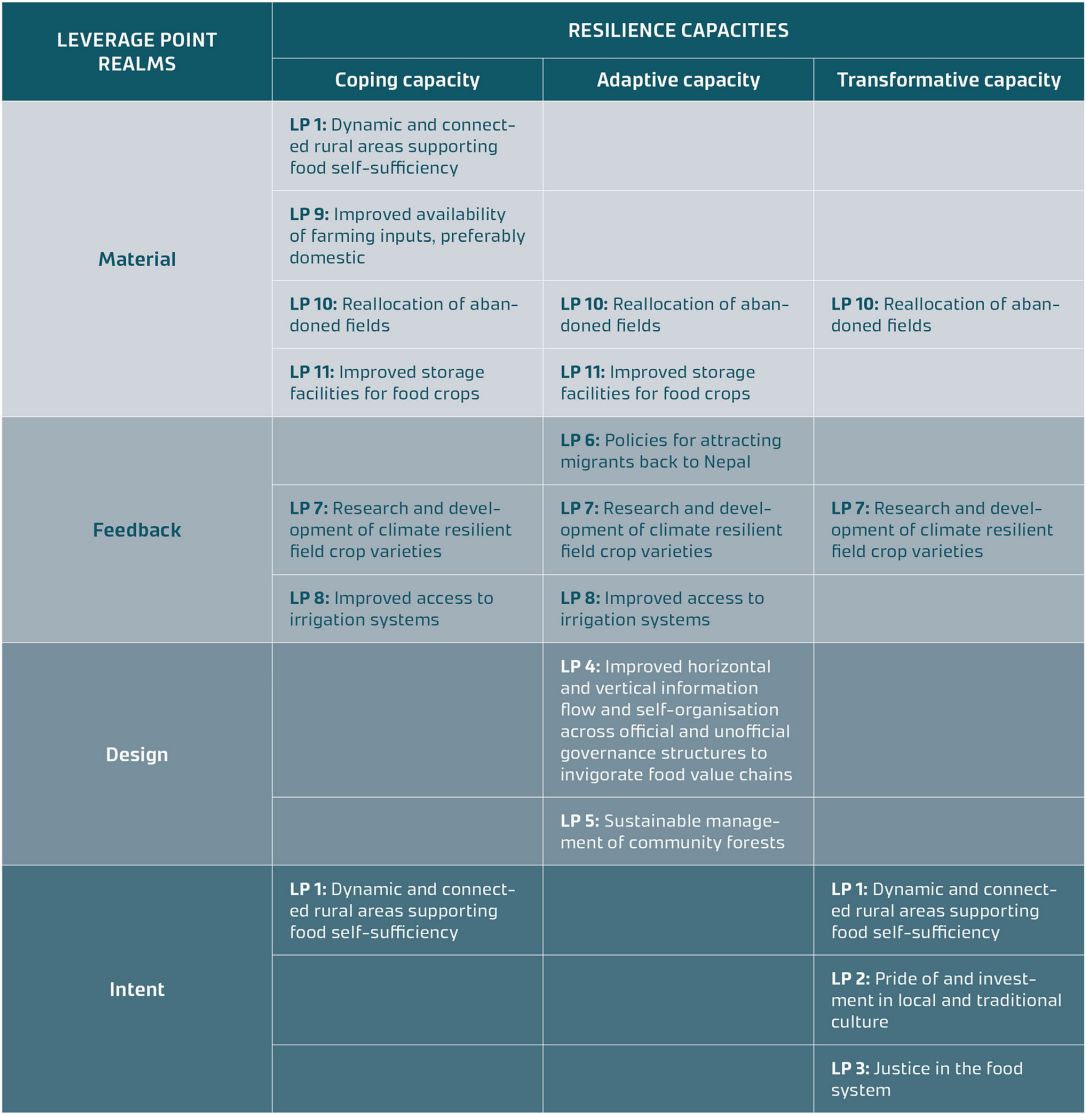
Summary of the eleven leverage points identified in Western Nepal and the intersection between the three resilience capacities (coping, adaptive and transformative capacity) and the four leverage point realms (intent, design, feedback and material). In some cases, the identified leverage points fit multiple intersections. The interpretations and justifications are presented in the Result section for each leverage point

#### Leverage point 2: Pride of and investment in local and traditional culture

The market economy and modern worldviews are gradually changing traditional cultures and mindsets in rural Nepal. Entire ways of life, such as that of the nomadic Raute hunter-gatherers in the High Mountains and Midhills, are on the verge of extinction despite government efforts to support their traditional lifestyle. Increased accessibility, facilitated by road infrastructure development, is bringing the market economy to previously isolated areas and altering traditional diets. While this transition has led to greater consumption of high-calorie, low-nutrition foods, such as instant noodles and chips, it has also improved access to vegetables and other healthy foods, which has reduced malnutrition and diversified diets.

Across the three agro-ecological zones—Terai, Midhills, and High Mountains—farming is widely perceived as a poor man’s occupation, a perception that is being passed down to the next generation. This belief is reflected in the words of a farmer in the High Mountains (key informant 34): *"There is no attraction towards farming in this generation. Most youths are going abroad, going to study in urban areas".*

Embracing traditional nutritious crops such as buckwheat, millet, and amaranth is crucial for improving nutrition, preserving cultural heritage, promoting sustainable agriculture, and strengthening resilient local economies. This approach not only supports rural communities but also fosters a more diverse, sustainable, and self-reliant food system in Nepal. Additionally, there is a growing interest for nutritious foods from the hills and mountains among the people living in urban areas, which presents a market opportunity.

Aligned with the intent realm, leverage point 2 (pride of and investment in local and traditional culture) addresses the mindset and paradigm from which the system arises (Fig. 1). It supports transformational capacity (Table 1) by fostering awareness and appreciation of the value and benefits of local and traditional culture, thereby opening alternative, positive futures to pursue.

#### Leverage point 3: Justice in the food system

Despite constitutional commitments to equality, western Nepal remains a deeply patriarchal society where lower-caste individuals continue to face discrimination and poverty. These entrenched inequalities are particularly pronounced in the Terai region, where patriarchal and discriminating values are embedded in the social fabric. Gender inequality and caste discrimination limit access to resources and hinder contributions to the food system.

Increased migration in search of work has resulted in remittances that improve the socio-economic status of the often lower-caste families who were previously excluded from salaried jobs. This development improves their social status locally. Migration is also slowly changing gender norms as the number of female-headed households increase as a result of departing males, enhancing women’s role and power in the family. However, men often remain the primary decision-makers, even from a distance, and women continue to rely on remittances for financial support. Migration has also increased women’s time poverty, as they now manage both household duties and farm labour.

Empowering women and marginalised communities through equitable policies, better access to resources, and increased participation in decision-making is still essential to unlocking the potential of Nepal’s agricultural sector and enhancing food security.

Leverage point 3 (Justice in the food system) challenges mindsets and the existing, dominant paradigm from which the system emerges, the intent realm (Fig. 1). Moreover, it enables transformative capacity (Table 1) by exposing existing injustices, fostering critical reflection, and envisioning alternative, more just futures. Additionally, it empowers marginalised communities by giving them the agency to pursue these changes.

### Addressing information flows and social structures—design realm

The study identified and contextualised two leverage points from the design realm: one concerning the food value chain and its organisation and the other regarding the rules of how community forestry is managed.

#### Leverage point 4: Improved horizontal and vertical information flow and self-organisation across official and unofficial governance structures to invigorate food value chains

Although connectivity through road construction and technological advancements like the internet and mobile phones are improving in western Nepal, social organisation and information flow within food value chains remain inadequate. The 2015 constitution introduced a devolved governance model, creating significant opportunities to transform Nepal’s food system through localised decision-making at the municipal level. Despite persistent challenges in capacity, coordination, and funding, the overall impact has, according to research participants, been positive and fostered a more responsive and inclusive agricultural sector. However, the food value chain still faces substantial obstacles that limit productivity and efficiency. Fragmented markets, often dominated by middlemen, squeeze profit margins for producers, while poor coordination across the value chain results in inefficiencies and missed opportunities for value-addition. This is reflected in the words of key informant 04 who says: “*When you travel up to Karnali itself, though people used to produce some products, they don’t have good linkages with the market. So, they don’t get good price. At the same time, we’re very aware that there’s a broker, and then the supplier, the farmers don’t get the real price of their products. And that is hindering “.*

Leverage point 4 (improved horizontal and vertical information flow and self-organisation across official and unofficial governance structures to invigorate food value chains) aligns with both the structure of information flows and the power to add, change, and self-organise in the design realm (Fig. 1). It enhances adaptive capacity (Table 1) by improving information flow through better communication channels, digital platforms, extension services, and transparent market systems. These are essential for increasing efficiency, reducing food loss, and empowering farmers to make informed decisions that promote agricultural growth and sustainability. Strengthening collaboration and information exchange among value chain actors is crucial for creating well-functioning value chains that can boost domestic food production and decrease reliance on farm input and food imports.

#### Leverage point 5: Sustainable management of community forests

Community forests contribute to Nepal’s food system by offering essential resources that support food security and livelihoods. These forests provide non-timber products such as fruits, nuts, and medicinal herbs, enhancing dietary diversity. Additionally, they serve as grazing areas for livestock, aid in soil and water conservation, and supply fuelwood for cooking. Despite their successes, Nepal’s community forests face challenges. One of these is linked to the strict policy of community forestry that prioritises conservation over sustainable management and use of forests. A better balance between conservation and sustainable forest management could support poverty reduction. Such a policy shift would, however, need to be done in a way that safeguards equitable benefit distribution, also benefitting marginalised community members.

Leverage point 5 (sustainable management of community forests) aligns with rules of the system in the design realm (see Fig. 1). A shift from a conservation-heavy focus to active, sustainable, and equitable forest management can support adaptive capacity through healthier ecosystems while providing food and livelihood opportunities for marginalised communities, promoting practices like agroforestry, and increasing access to the collection of non-timber forest products (NTFP), such as berries and mushrooms (Table 1).

### Addressing migration, climate change impacts, and the water systems through system feedbacks—feedback realm

The study addresses three key feedback mechanisms that notably affect Nepal’s food system: migration, climate change’s impact on crop yields, and availability of water in farming systems.

#### Leverage point 6: Policies for attracting migrants back to Nepal

Migration is an increasingly significant socioeconomic phenomenon impacting Nepalese socio-ecological system at multiple levels. While migration provides income through remittances, it also depletes the country of dynamic, young individuals who are essential for driving economic development. Migration profoundly influences Nepal’s macroeconomic landscape, with remittances contributing around 25% of national GDP (Banjara et al. 2020). These inflows stabilise the economy, support household consumption, and bolster foreign reserves. However, this reliance on external economies creates vulnerabilities. Out-migration results in labour shortages in critical sectors like agriculture, driving up local wages and reducing productivity. This is reflected in the comments of key informant 07: *“It’s more expensive to plow with oxen in Nepal than anywhere else. People who plow with oxen may earn around 1500 in a day”.* Key informant (29) says: *“It’s hard to find labourers when you need them. Usually, neighbours help each other out once they finish their own work”.* Additionally, the loss of skilled labour contributes to a "brain drain", weakening essential sectors such as healthcare and education.

While remittances can drive investment and support families, e.g. by enabling children’s education, investments in local productive ventures remain limited, possibly hindering long-term economic diversification. Increased consumption can also lead to inflation, especially in housing and essential goods, putting financial pressure on households without remittances. Despite offering crucial economic support, Nepal’s dependence on remittances makes the country vulnerable to external economic shocks, highlighting the need for local economic development and diversification for sustainable growth.

From a systems thinking perspective, people in this context are the system stock and migration represents a system flow. As more Nepalese leave the country, this trend accelerates, with earlier migrants paving the way for others, creating a positive feedback loop that speeds up migration. Leverage point 6 (policies for attracting migrants back to Nepal) aims to counteract this (Fig. 1). It strengthens adaptive capacity (Table 1) as capable and dynamic individuals from diverse professional backgrounds who return to Nepal can provide important new innovative ideas, revitalising the business and social environment. Returning migrants can also improve the situation in sectors that are currently suffering from a shortage of workforce, and lower redundancy.

#### Leverage point 7: Research and development of climate-resilient field crop varieties

Climate change is affecting Nepal’s food system, particularly crop yields. Rainfall patterns have become more unpredictable and extreme, causing both prolonged droughts and floods. Rapid glacial melting in the Himalayas increases the risk of flooding, endangering lives, and infrastructure. Climate-resilient crops can withstand these stresses, ensuring more stable production even in adverse conditions. Resilient varieties can also help reduce dependency on food imports. Adapting to climate change is inherently specific to local contexts, which means that research and development of new climate-resilient field crop varieties must be tested in Nepalese conditions, if not be explicitly tailored to meet the country’s needs. As key informant 04 says: “*In India, they have developed a lot of climate-resilient seeds for the cold. But they have developed the seeds which are suitable to their environment. People like us, we keep on procuring, or our suppliers keep on supplying the seeds to the Middle hill or the Upper hill which is completely unsuitable for that particular environment”.*

Leverage point 7 (research and development of climate-resilient field crop varieties) highlights the need for more research into diverse, traditional crops and their varieties, which hold potential for offering higher farm system resilience through increased diversity. It addresses climate change, which acts as a positive feedback loop (Fig. 1), accelerating crop failures and deepening the hardship of Nepalese farmers. The leverage point supports coping, adaptive, and transformative capacity (Table 1) in distinct ways: coping capacity, by preserving a diverse range of field crop varieties, this approach helps safeguard food security during floods and droughts. It supports adaptive capacity through ongoing research and development of climate-resilient crop varieties to ensure the continued viability of farming in Nepal. Finally, it supports transformative capacity by fostering a renewed appreciation for traditional crops, which have been locally adapted for centuries; this approach strengthens long-term resilience and sustainability.

#### Leverage point 8: Improved access to irrigation systems

The availability of sufficient water outside the monsoon season is a major bottleneck in the Nepalese farming system. With climate change posing additional stress on water availability, the importance of well-functioning and available irrigation systems will become increasingly important to the success or failure of the Nepalese farming system. This is reflected in the words of key informant *11, who says: “Water availability is our main problem. Even now we do not have sufficient water lines. We have understood that if we have water, we can achieve our goals”.* Leverage point 8 (improved access to irrigation systems) thus addresses a positive feedback loop (Fig. 1) caused by climate change and supports coping capacity (Table 1) by buffering the shortage of water availability during droughts and supporting adaptive capacity as a longer-term adaptive water management strategy.

### Addressing food system stocks and flows—material realm

The material realm represents the most superficial level of the leverage point framework, addressing system stocks and flows. In the context of the western Nepalese food system, the study identified two leverage points relating to system stocks: one relating to reallocation of abandoned farmland and the other to the availability of agricultural inputs. In terms of system flows, the study identified a leverage point relating to post-harvest storage facilities.

#### Leverage point 9: Improved availability of farming inputs, preferably domestic

The limited availability of farming inputs, such as seeds and fertilisers, acts as a significant bottleneck in the Nepalese food system. The mechanisation level of the farming sector is also low. This scarcity of farming inputs constrains agricultural productivity, negatively affecting food security and the livelihoods of farmers. Nepal heavily relies on imported farming inputs, and the domestic production of commercial fertilisers and seeds is minimal, leaving the country vulnerable to external trade and political shocks. Enhancing the availability of domestic farming inputs could help secure a more stable supply of these essential products.

Leverage point 9 (improved availability of preferably domestic farming inputs) aligns with the material realm and the structure of material stocks and flows (Fig. 1). It improves coping capacity (Table 1) by safeguarding against trade disruptions and climate shocks that can restrict access to farming inputs directly impacting food security. It can also be interpreted as improving adaptive capacity by offering a longer-term safeguarding mechanism building resilience through improved redundancy and diversification of farming inputs.

#### Leverage point 10: Reallocation of abandoned fields

Large-scale migration from the hills and mountains has resulted in abandonment of land previously dedicated to crop production. Many of these areas are challenging to farm due to their remoteness or low fertility. To support food security, food self-sufficiency, and livelihood opportunities, it would be beneficial to explore schemes that facilitate the reallocation of these abandoned fields. As the limited availability of labour in farming can be a bottleneck, the reallocation should be done in a way that requires less labour than before. For instance, in some cases, these lands could be used for fruit production, such as apple cultivation. Additionally, it could be viable to expand livestock production as these abandoned fields offer a potential grazing source for goats and cows. Reallocation of land user rights to neighbours who are willing to cultivate the lands is another option for these fields.

Leverage point 10 (reallocation of abandoned fields) aligns with the material realm and more precisely the structure of material stocks and flows (Fig. 1). Through this interpretation, it improves both coping and adaptive capacity (Table 1) by protecting against trade disruptions and climate shocks that can lead to crop failure impacting food security (coping capacity) and offering a longer-term safeguarding mechanism (adaptive capacity). Leverage point 10 can also be seen to align with the deepest leverage point realm, the intent realm. According to this interpretation, it supports transformative capacity by granting farmlands to landless, marginalised community members and hence supporting justice.

Leverage point 11: Improved storage facilities for food crops

Storage facilities in western Nepal are underdeveloped, creating significant challenges for the food value chain as many crops, particularly fruits and vegetables, are prone to spoilage. Food loss due to inadequate storage is a critical issue impacting food security, farmer incomes, and the broader economy. Investment in enhanced storage infrastructure is essential, along with the adoption of modern storage technologies. Additionally, providing capacity building for farmers on post-harvest management and improving access to cold chain facilities will help preserve perishable produce. As key informant 10 points out: *“I believe it would be beneficial if Bheriganga Municipality or the government could establish storage depots. Currently, there are no such facilities. In times of crisis, people would only be able to rely on what they have stored themselves. There is no organized support from any institutions”.*

**Fig. 4:**
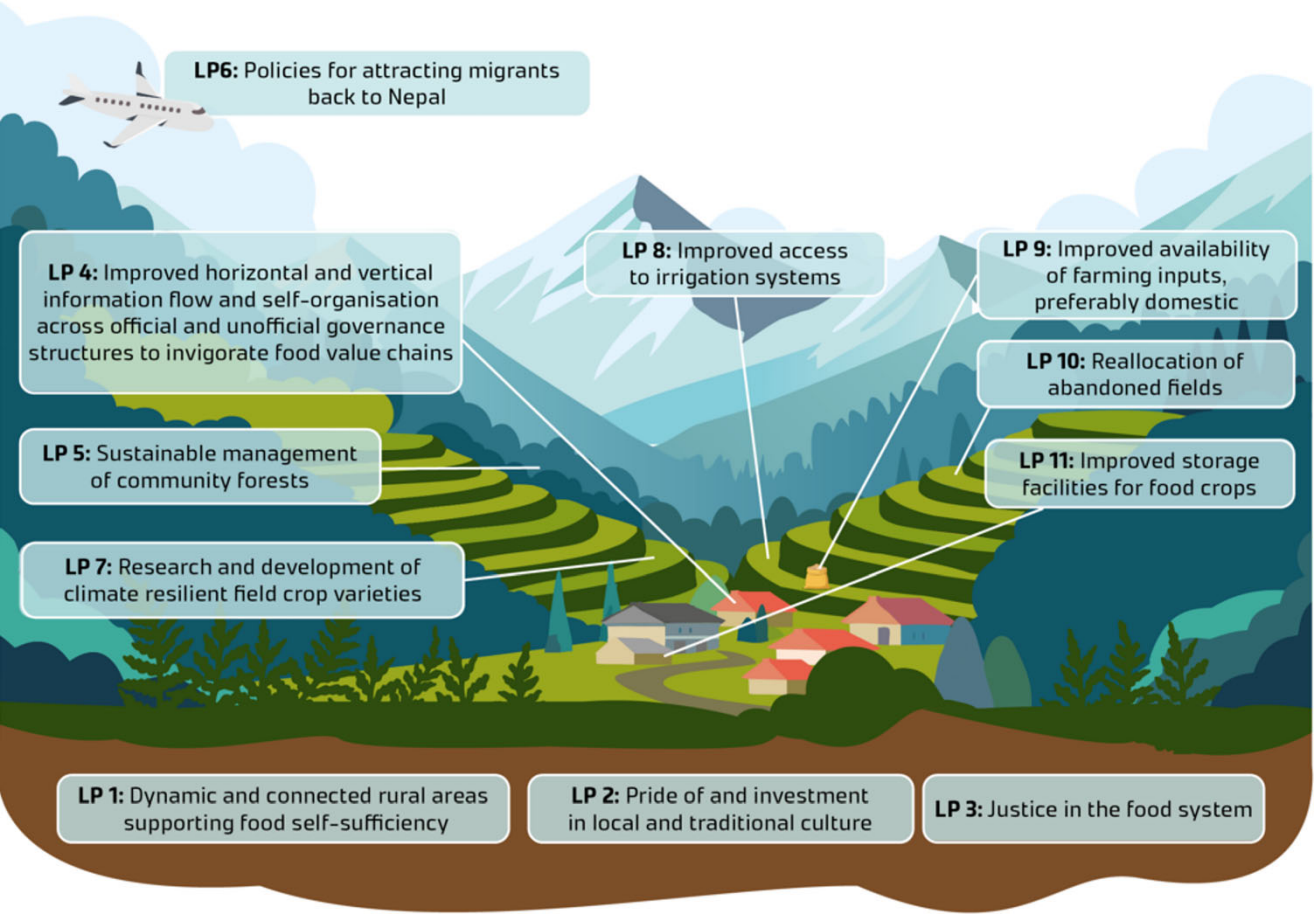
Summary of the identified leverage points in Western Nepal

Leverage point 11 aligns with the size of the buffer stocks, relative to the flows (Fig. 1). It enhances coping capacity (Table 1) in time of crises but also adaptive capacity in terms of offering a long-term safeguarding mechanism (Fig. 4).

## Discussion

The objective of this study was to design and apply a theoretical approach combining the leverage point framework with the three resilience capacities: coping, adaptive, and transformative capacity. This so-called pyramid framework (Fig. 1) aims to shed light on the root causes that shape resilience and identify effective entry points to strengthen resilience and map transformative pathways. Below we discuss the results of applying the approach to an assessment of the food system in western Nepal and deliberate on the benefits and possible implications for policy and practice.

### Reflections on the results from Nepal

When aligning the four realms of the leverage point framework (intent, design, feedback, and material) with the three resilience capacities, a clear analogy emerges: Intent realm aligns with transformative capacity, design realm aligns with adaptive capacity, and feedback and material realms align with coping capacity. To test this hypothesis, we applied the pyramid framework in western Nepal.

The results support the hypothesis as all the three identified leverage points from the intent realm support transformative capacity. Both design realm leverage points support adaptive capacity. Two out of three of the feedback leverage points support coping capacity, and all three leverage points identified from the material realm support coping capacity.

But the results add important nuance to the analysis. For example, Leverage point 10 (reallocation of abandoned fields) is classified in the study as a “shallow” leverage point in the material realm as it concerns a system stock (fields). Yet, reallocating these fields to ultra-poor, landless, and marginalised communities can enable deeper systemic change. On the other end of the spectrum, Leverage point 1 (dynamic and connected rural areas supporting food self-sufficiency), which sits in the intent realm was classified as supporting both transformative capacity—due to the broader ambition to revitalise rural areas—but also coping capacity as food self-sufficiency safeguards against trade disruptions and climate shocks. All of the identified leverage points of the material and feedback realms, with one exception (Leverage point 9: Improved availability of farming inputs, preferably domestic), were found to support both coping and adaptive capacity. Leverage point 9 only supported coping capacity. When classified as supporting adaptive capacity, they contribute to long-term resilience strategies, whereas when categorised under coping capacity, they serve as immediate safeguards against external shocks such as trade disruptions and climate variability.

### Advantages of the pyramid framework

The pyramid framework comprises two primary sides: one representing the leverage points framework and the other depicting the three resilience capacities. The resilience capacity side emphasises the need to consider coping, adaptive, and transformative capacities simultaneously. Meanwhile, the leverage points side helps identify the root causes shaping resilience and their connections to different types of resilience capacities.

A key distinction of the pyramid framework—compared to approaches such as the ABCD food system resilience framework (Fonteijn 2022) or the TARA approach (Colloff et al. 2017)—is that it simultaneously integrates all three resilience capacities. It considers responses to varying levels of shock intensity over different time scales, addressing both immediate reactions and long-term strategic planning. Prior research has shown that relying too heavily on a single capacity, such as coping strategies, can reduce resilience over time (Béné et al. 2016), which is a key concern in maladaptation literature (Juhola et al. 2016; Magnan et al. 2016; Schipper 2020). By acknowledging the interconnections between resilience capacities, the framework helps identify and mitigate trade-offs between them.

Additionally, the leverage points framework serves as a guide for identifying effective entry points to strengthen resilience. The framework encourages reflection around both shallow and deep aspects. It is perhaps particularly helpful for addressing underlying paradigms, social norms, power structures, and systemic goals—elements that according to critical theory and leverage point theory are essential to preventing the reproduction of oppressive structures and achieving deep, lasting transformation (Meadows 1999; Devetak 2022). This is why addressing deep leverage points, such as leverage point 3 *(justice in the food system)* is critical.

Achieving transformative change is difficult due to hegemonic power structures and path-dependent system lock-ins, where past decisions create self-reinforcing mechanisms that resist change (Simoens et al. 2022). Overcoming these barriers may require disrupting the system (Geels 2018; Morrison et al. 2022) and unlearning entrenched knowledge and practices (Tsang and Zahra 2008; Bellandi et al. 2021). Transformative knock-on effects, where small shifts trigger broader systemic transformation, offer another pathway for change. While system transformation is a key policy goal at national and international levels, clear roadmaps for achieving it are often lacking. We argue that the pyramid framework can serve as a tool for identifying transformative pathways for strengthening resilience by identifying leverage points at different depths with complementary and catalytic functions.

Resilience theory suggests that overemphasising coping and adaptive capacities may hinder deeper transformation (Carr 2019). However, leverage points theory proposes that shallower leverage points—such as material or feedback-level changes—can catalyse deeper systemic shifts (Rosengren et al. 2023). This concept, known as a "chain of leverage" (Fischer and Riechers 2019), provides a structured approach for identifying transformative pathways. For example, in Nepal, Leverage Point 9 (improved availability of farming inputs)—a shallow, material leverage point) supports Leverage Point 1 (dynamic and connected rural areas supporting food self-sufficiency). In turn, Leverage Point 1 can be reinforced by Leverage Point 4 (improved horizontal and vertical information flow and self-organisation across governance structures), which strengthens food value chains.

Finally, the pyramid framework’s flexibility in scale is a key advantage. We recommend further testing its applicability across both large-scale systems such as entire regions and on a smaller system in individual communities to refine its potential for guiding resilience-building and transformative efforts.

### Implication for policy and practice

From a policy perspective, the pyramid framework (Fig. 1) serves as a strategic tool for guiding adaptation planning and resource allocation. It can also be used to assess and evaluate existing resilience policies and interventions. Its flexibility may allow for application across different jurisdictional scales. The pyramid’s structure underscores the importance of prioritising deeper aspects of resilience-building, as these foundational elements shape social structures and drive systemic change. While these deeper aspects may be challenging to quantify, they remain crucial for achieving lasting resilience and should not be overlooked.

The leverage points side of the pyramid, with its 12 different leverage point types, informs adaptation planning by guiding the identification of strategic entry points for strengthening resilience. The three resilience capacities—coping, adaptive, and transformative—ensure a nuanced approach that considers both the reactive and forward-looking aspects of resilience.

For practitioners, the framework serves as a clear and accessible tool that can function as a boundary object (Ungar 2018; Fischer and Riechers 2019), meaning a shared reference point that fosters communication and collaboration among diverse social groups. This enables collective efforts to strengthen resilience, even when stakeholders have different perspectives or objectives.

Further refinement of the pyramid framework could enhance its practical application. For instance, developing a set of guiding questions could help practitioners reflect on strategies to reinforce resilience and navigate transformative pathways more effectively.

## Conclusion

The application of the pyramid framework in western Nepal highlights a clear relationship between deep leverage points and transformative capacity, as well as between shallow leverage points and coping or adaptive capacity. Integrating the leverage point framework with the three resilience capacities—coping, adaptive, and transformative—proves valuable for identifying effective entry points to strengthen resilience. This includes both reactive measures and forward-looking strategies.

The framework also brings to light underlying system drivers—such as paradigms, assumptions, values, worldviews, and social structures—fostering critical reflection and facilitating improvements, particularly in transformative capacity. By addressing all three resilience capacities simultaneously, the pyramid framework balances immediate responses to shocks with long-term planning. This integrated approach helps anticipate and mitigate trade-offs between coping, adaptive, and transformative strategies, reducing the risk of maladaptation that could undermine resilience over time.

Key deep leverage points identified in this study include redefining food system goals to enhance self-sufficiency and promoting cultural pride in local and traditional food and farming practices. While addressing these deep-seated factors is critical for fostering transformative capacity, shallow leverage points also play a vital role. These include practical, tangible actions such as improving access to domestic farming inputs, reallocating farmland, and upgrading irrigation systems—entry points that can pave the way for deeper systemic transformation.

The leverage points identified offer a concrete example of food system transformation in a Global South context, helping to operationalise the often abstract concept of transformation. Although this study focuses on western Nepal’s food system, the framework may be more broadly applicable to food systems in both the Global South and North. Moreover, its flexibility suggests potential for adaptation to other sectors, such as energy and water systems—areas that warrant further exploration.

These insights offer valuable guidance for policymakers, government officials, and NGOs, highlighting priority areas for effective policy development and strategic resilience planning. The pyramid framework serves as a practical tool for both identifying effective entry points and transformative pathways and for assessing and evaluating existing resilience policies and interventions. Its scalability across different systems and contexts, along with its ability to foster communication among diverse stakeholders, makes it a promising approach for enabling collective efforts to strengthen resilience.

## Data Availability

Data cannot be shared openly but are available on request from authors.
